# Dataset of Edmonds’ bi-vectors and tri-vectors with realizations

**DOI:** 10.1016/j.dib.2024.110785

**Published:** 2024-07-31

**Authors:** Endre Boros, Vladimir Gurvich, Matjaž Krnc, Martin Milanič, Jernej Vičič

**Affiliations:** a RUTCOR, Rutgers University, USA; b National Research University Higher School of Economics (HSE), Moscow, Russia; c University of Primorska, FAMNIT, Glagoljaška 8, 6000 Koper, Slovenia; d University of Primorska, IAM, Muzejski trg 2, 6000 Koper, Slovenia; e Research Centre of the Slovenian Academy of Sciences and Arts, The Fran Ramovš Institute, Novi trg 2, 1000 Ljubljana, Slovenia

**Keywords:** Embedding dual graphs into surfaces, Triangulations, Dual maps, Edmonds’ property, Bi-vectors, Tri-vectors

## Abstract

In 1965, Jack Edmonds characterized pairs of graphs **G** and G* with a bijection between their edge sets that form a pair of dual graphs realizing the vertices and countries of a map embedded in a surface. A necessary condition is that, if **d** = (d_1_, …, d_n_) and **t** = (t_1_,…, t_m_) denote the degree sequences of two such graphs, then $\sum_{i=1}^{n}d_{i}=\sum_{j=1}^{m}t_{j}=2l$, where l is the number of edges in each of the two graphs and χ=n+m−l is the Euler characteristic of the surface. However, this condition is not sufficient, and it is an open question to characterize bi-vectors (**d, t**) that are **geographic**, that is, that can be realized as the degree sequences of pairs **G** and G* of surface-embedded graphs.

The above question is a special case of the following one. A multigraph **G** is even if each vertex has even degree and 3-colored if **G** is equipped with a fixed proper coloring of its vertex set assigning each vertex a color in the set {1,2,3}. Let **G** be a 3-colored even multigraph embedded in a surface **S** so that every face is a triangle. Denote by **d** = (d_1_, …, d_n_), **t** = (t_1_, …, t_m_), and **δ** = (δ_1_, ..., …, δ_k_) the sequences of half-degrees of vertices of **G** of colors 1, 2, and 3, respectively. Then, $\sum_{i=1}^{n}d_{i}=\sum_{j=1}^{m}t_{j}=\sum_{\mu =1}^{k}t_{\mu }=l$, where χ=n+k+m−l is the Euler characteristic of the surface **S.** A tri-vector **(d, t, δ)** satisfying the above conditions is called **feasible**. A feasible tri-vector is called **geographic** if it is realized by a 3-colored triangulation of a surface. Geographic tri-vectors extend the concept of geographic bi-vectors.

We present a dataset of geographic bi-vectors and tri-vectors, along with realizations proving that they are geographic.

Specifications Table

**Table:** 

Subject	Mathematics
Specific subject area	Discrete Mathematics and Combinatorics Geometry and Topology
Data format	Analyzed
Type of data	Table
Data collection	The data for this dataset were computed using two ad-hoc programs implementing two comparable, but different algorithms, both presented in Section Experimental design, materials and methods. The first algorithm was implemented in Java and executed on a node of the computing cluster at UP FAMNIT (AMD Ryzen Threadripper 1950×16-Core Processor). The second algorithm was executed on one kernel of the COCALC installation at UP FAMNIT (Intel e5 2699v4 processor).
Data source location	University of Primorska, UPFAMNIT, Glagoljaška 8, 6000 Koper, Slovenia
Data accessibility	Repository name: Zenodo Dataset name: Dataset of Feasible, Edmonds' and Geographic Bi-vectors and Tri-vectors [1] Data identification number: DOI:10.5281/zenodo.8032314 Direct URL to data: https://doi.org/10.5281/zenodo.8032314
Related research article	

## 1 Value of the Data

The presented dataset of Edmonds’ vectors with all Edmonds’ realizations holds significant value for a range of research projects:

• These data are useful for research dealing with embedding dual graphs on surfaces, an interdisciplinary research area at the intersection of graph theory and topology. Furthermore, the dataset can be used in industry or art to design/create new surfaces.
• The following are the identified potential users of the data: topologists and graph theorists dealing with various topological surfaces and maps on them, artists, and engineers.
• Using the attached script, the JSON files from the dataset can be imported to obtain bi-matrices, which give rise to the corresponding dual graphs and thus bypassing the time-consuming step of discovering Edmonds’ realizations for a selected range of bi- and tri-vectors.

## 2 Background

The objective of this paper is to provide new useful tools for surface designers; namely, bi-matrices witnessing Edmonds’ realization for all bi- and tri-vectors. See technical details in [[2], [3],^4^]. The preprinted paper Boros et al., [4] explains the process and the rationales that produced presented dataset in detail.

Those witnessing bi-matrices are important for discovering new patterns and theoretical insights regarding bi-vectors and tri-vectors realizable in a surface. Indeed, the data already enabled the present authors to obtain several new theoretical insights in the respective subfield of topology and graph theory.

## 3 Data Description

The dataset consists of two sets of files in JSON format. Each file consists of an array of elements and each element is presented by a vector, more precisely, a bi-vector in the case of the first set, and a tri-vector in the second case. This vector is followed by the Euler characteristic of the surface and a set of Edmonds’ realizations, described as a concatenation of two incidence matrices. Such a bi-matrix gives rise to the corresponding graph, its dual on the surface, as well as a bijective correspondence between their edge sets.

**Fig. 1:**
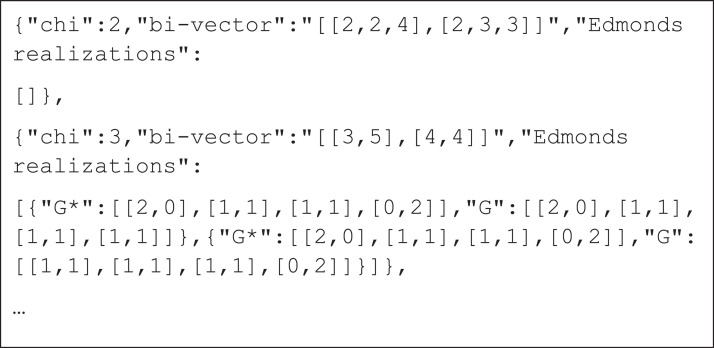
A section of the file describing bi-vectors. The file stores bi-vectors in JSON array, each element defines a bi-vector with all feasible Edmonds’ realizations stored in bi-matrices. Each bi-matrix defines a graph G and a dual graph G*, both stored in the same named key-value pairs. The first bi-vector ([[2,2,4],[2,3,3]]) has no Edmonds’ realizations.

The first set consists of 6 files, each containing all possible bi-vectors with all Edmonds’ realizations in a form of bi-matrices for a fixed l (ranging from 2 to 7). Fig. 1 shows a section of the file for l=4.

The bi-vectors are stored in files with the value of *l* mentioned at the end:

feasible-edmonds-bi-vector-realization-ell2.json

feasible-edmonds-bi-vector-realization-ell3.json

feasible-edmonds-bi-vector-realization-ell4.json

feasible-edmonds-bi-vector-realization-ell5.json

feasible-edmonds-bi-vector-realization-ell6.json

feasible-edmonds-bi-vector-realization-ell7.json

**Fig. 2:**
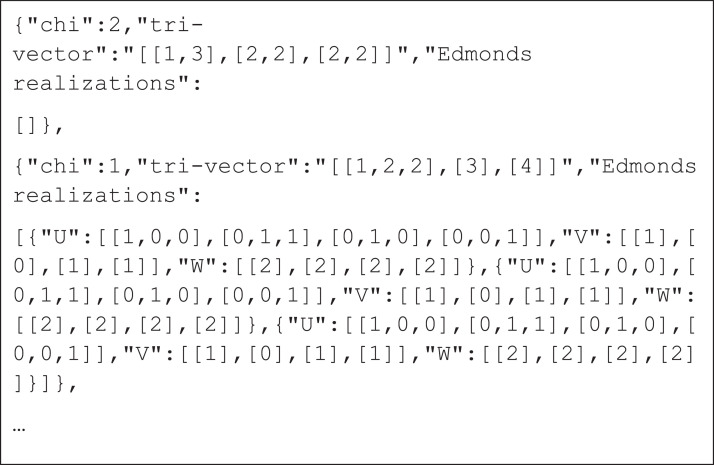
A section of the file describing tri-vectors. The file stores tri-vectors in JSON array, each element defines a tri-vector with all feasible Edmonds’ realizations (only one for l=8,9) stored in tri-matrices. The first two parts of each tri-matrix define a bipartite graph (each part is stored in a separate matrix called U and V, respectively). The third part of the trimatrix (matrix W) represents the corresponding dual graph. All three parts of the tri-matrix are stored in the same named key-value pairs. Note that the tri-vector ([[1,3],[2,2],[2,2]]) has no Edmonds’ realizations.

The second set consists of 8 files, each containing all possible tri-vectors with all Edmonds’ realizations in a form of bi-matrices for a fixed l (ranging from 2 to 7). The last two files (for l equal to 8 and 9 respectively), contain only one Edmonds’ realization due to time complexity. Fig. 2 shows a section of the file for the case l=4.

The tri-vectors are stored in files with the value of *l* mentioned at the end:

feasible-edmonds-geographic-vector-realization-ell2.json

feasible-edmonds-geographic-vector-realization-ell3.json

feasible-edmonds-geographic-vector-realization-ell4.json

feasible-edmonds-geographic-vector-realization-ell5.json

feasible-edmonds-geographic-vector-realization-ell6.json

feasible-edmonds-geographic-vector-realization-ell7.json

feasible-edmonds-geographic-vector-realization-ell8.json

feasible-edmonds-geographic-vector-realization-ell9.json

A set of utilities show use cases:

[1] Parsing functions with examples implemented in Sage: edmonds_import.sage
[2] Parsing functions with examples implemented in Java:
  EdmondsReaderBivector.java
  EdmondsReader.java
[3] Usage examples in make utility:
  Makefile

## 4 Experimental Design, Materials and Methods

The data for this dataset was computed using two programs implemented using different technologies and implementing different algorithms. Both algorithms search through the whole space of feasible vectors.

• The first algorithm checks all Edmonds’ realizations.
• The second algorithm stops at the first found Edmonds’ realization, thus being able to search bigger search space but presenting just one of the possible realizations.

One of the purposes of this approach was to be able to search for the vector examples that are feasible, but not Edmonds’ realizable, and the other was to cross-compare the results to eliminate errors in implementation, thus ensuring reproducibility.

The selection of the underlying technologies and programming languages was purely pragmatic (availability and familiarity). The first algorithm was implemented in Java and executed on a node of the computing cluster at UP FAMNIT (AMD Ryzen Threadripper 1950×16-Core Processor). The second algorithm was executed on SAGE [5] installation at UP FAMNIT (Intel e5 2699v4 processor). The time complexities of the presented algorithms cannot be compared to each other, as one was used to collect all possible Edmonds’ realizations and the other stops at the first evidence of a valid Edmonds’ bi-matrix.

## Limitations

The lists are not exhaustive as the number of edges in pairs of dual graphs may be arbitrarily large. The used algorithms allow to fully search only small graphs (up to 9 edges in our dataset).

## Ethics Statement

The authors have read and follow the ethical requirements for publication in Data in Brief and confirming that the current work does not involve human subjects, animal experiments, or any data collected from social media platforms.

## CRediT authorship contribution statement

**Endre Boros:** Conceptualization, Software, Investigation, Supervision, Writing – review & editing, Data curation. **Vladimir Gurvich:** Conceptualization, Software, Investigation, Supervision, Writing – review & editing, Data curation. **Matjaž Krnc:** Conceptualization, Software, Investigation, Supervision, Writing – review & editing, Data curation. **Martin Milanič:** Conceptualization, Software, Investigation, Supervision, Writing – review & editing, Data curation. **Jernej Vičič:** Conceptualization, Software, Investigation, Supervision, Writing – review & editing, Data curation.

## Data Availability

Dataset of Feasible, Edmonds' and Geographic Bi-vectors and Tri-vectors (Original data) (Zenodo)
